# Benefit of salvage total pharyngolaryngoesophagectomy for recurrent locally advanced head and neck cancer after radiotherapy

**DOI:** 10.1186/s13014-017-0900-2

**Published:** 2017-10-26

**Authors:** Jie Liu, Ye Zhang, Zhengjiang Li, Shaoyan Liu, Huizheng Li, Zhengang Xu

**Affiliations:** 1 Department of Head and Neck Surgical Oncology, National Cancer Center/Cancer Hospital, Chinese Academy of Medical Sciences, Peking Union Medical College, No 17 Panjiayuan Nanli, Chaoyang District, Head and Neck Ward 1, Beijing, 100021 China; 2 Department of Radiation Oncology, National Cancer Center/Cancer Hospital, Chinese Academy of Medical Sciences, Peking Union Medical College, Beijing, China; 3 Department of Otorhinolaryngology, Dalian Friendship Hospital, Dalian, China

## Abstract

**Background:**

The treatment modalities for recurrent locally advanced head and neck cancer failure after radiotherapy are limited with poor prognosis. Salvage supra-radical operation seems to be an option. It has not been established which patients will benefit from salvage total pharyngolaryngoesophagectomy.

**Methods:**

We retrospectively reviewed 66 patients with previously irradiated recurrent T4 head and neck cancer who underwent salvage total pharyngolaryngoesophagectomy at our institution between January 2001 and June 2014. The clinical outcome and toxicities were analyzed.

**Results:**

Flap loss occurred in 2 patients, and the incidence of fistulas and anastomosis strictures was 15.6% (10/66) and 13.6% (9/66), respectively. The median survival time of the entire cohort was 12 months. The interval between radiation and salvage surgery, and microscopic carotid artery invasion were identified as independent prognostic factors for overall survival. The 3-year overall survival rates of patients with (*n* = 33) and without (*n* = 33) risk factors were 9.1% and 47.2%, respectively (*p* = 0.007). A time interval between radiation and salvage surgery ≤6 months and previous concurrent chemotherapy or targeted therapy were risk factors for severe post-operative complications.

**Conclusions:**

Salvage total pharyngolaryngoesophagectomy is beneficial to selected patients with recurrent locally advanced head and neck cancer after radiotherapy.

## Introduction

The effective treatment for recurrent locally advanced head and neck cancers after radiotherapy is limited. The median survival of patients who received chemotherapy as the standard treatment is 6–9 months [1]. The majority of patients have a high rate of local failure after non-surgical treatment, especially in recurrent stage T4 patients [2–4]. It has been reported that salvage aggressive surgery might lead to long-term survival in these patients [2].

Total pharyngolaryngoesophagectomy (TPLE) has been confirmed technically feasible and effective, and could achieve long survival for the recurrent locally advanced head and neck patients [2]. TPLE coupled with upper digestive tract reconstruction also expand the extent of the salvage surgery with better quality of life (QOL). Considering that few treatment modalities can be appropriate for recurrent locally advanced head and neck cancer, TPLE coupled with upper digestive tract reconstruction has been attempted on these patients as an option.

The purpose of the current study was to assess the comparative efficacy of salvage TPLE for previously irradiated, recurrent T4 head and neck cancer, and to identify the factors that affect the prognosis and complications. Our study could help surgeons identify which group of patients could most likely benefit from this supra-radical surgical salvage strategy.

## Patients and methods

### Patient selection

Between January 2001 and June 2014, a total of 66 patients who underwent salvage TPLE in the Department of Head and Neck Surgical Oncology in our hospital were retrospectively reviewed. Before initiation of this study, Institutional Review Board approval was obtained. The selection criteria were as follows: (1) squamous cell carcinoma of the head and neck cancer; (2) recurrent locally advanced head and neck cancer (stage rT4 according to the seventh AJCC stage system); (3) definitive/adjuvant radiotherapy was performed in the previous treatment with a dose >50 Gy; (4) clinical rT4b cases was excluded; and (5) all patients declined chemotherapy. Patients were evaluated pre-operatively using endoscopy, abdominal ultrasonography, chest computed tomography (CT), and head and neck magnetic resonance imaging (MRI). Medical records were retrospectively analyzed to gather the clinical characteristics and surgical outcomes.

### Operative techniques

TPLE or completion of TPLE and microvascular free tissue transfer were performed on all cases. The donor site for digestive reconstruction included free jejunum (*n* = 63) and free flaps, including 2 anterior lateral thigh flaps and 1 forearm flap, and 12 pectoralis major myocutaneous flaps and 2 ALT flaps were also used for skin reconstruction on 14 patients with skin invasion. The surgical procedure was under a standard step. Tumor resection and flap elevation were performed by two teams simultaneously. TPLE always followed the *en bloc* principle; specifically, the cervical sheath was separated bilaterally with the specimen first and the thyroid gland was generally removed if suspicious invasion was detected. Transection of the trachea was performed, and separated from the cervical esophagus and oropharynx. The cervical esophagus was also transected at the proper level according to the extent of tumor. Surgical margins were examined with frozen sections until clean margins were achieved if possible. The jejunum segment or flap tube was placed to repair the digestive tract. After complete enteric anastomosis, the microscopic vascular anastomoses were established. Finally, irrigation and hemostasis, a drain-setting permanent tracheostomy, and skin closure were performed.

### Statistics

All of the patients were followed until June 2015. Kaplan-Meier curves were used to evaluate the overall survival of the cohort. Multivariate analysis was performed to identify the possible risk factors of poor prognosis and bilateral logistic regression was used to evaluate the factors of severe complications. All statistics were performed using.

SPSS 17.0 (SPSS, Inc., Chicago, IL, USA).

## Results

### Patient status before salvage surgery

The characteristics of the entire cohort are presented in Table 1. Of the 66 patients, 5 were females, whereas the remaining 61 patients were males with a mean age of 58.6 years (age range, 42–81 years) at the time of salvage surgery. The primary sites were laryngeal cancer in 19 patients, hypo-pharyngeal cancer in 40 patients, and cervical esophageal cancer in 7 patients. Primary treatment varied according to the site of lesions. Surgery was the main initial treatment for patients (17/19) with laryngeal cancer; approximately one-half had two or more previous surgeries. Radiotherapy or concurrent chemoradiation was mostly used as the first treatment in patients with hypo-pharyngeal (32/40) and cervical esophageal (7/7) cancers. Of the entire cohort, 51 patients underwent radical radiotherapy. In the remaining 15 patients, radiation was performed post-operatively. The radiation dose ranged from 50 to 70 Gy, and 20 patients underwent concurrent chemotherapy and/or targeted therapy.

**Table 1:** Characteristics of cases before salvage surgery

Characteristics	n	%
Age in years		
> 60	30	45.5%
≤ 60	36	54.5%
Primary		
Larynx	19	28.8%
Hypo-pharynx	40	60.6%
Cervical esophagus	7	10.6%
Previous treatment		
1 course without surgery	30	45.5%
1 course with surgery	26	39.4%
Multiple courses	10	15.2%
Radiation dose		
≤ 60Gy	14	21.2%
> 60Gy	52	78.8%
Time interval between radiation and salvage surgery		
≤ 6	14	21.2%
> 6	52	78.8%
Previous chemo- or targeted therapy		
No	46	69.7%
Yes	20	30.3%
Skin invasion		
No	52	78.8%
Yes	14	21.2%
Pre-op suspicious carotid artery invasion^a^		
No	53	80.3%
Yes	13	19.7%
Pre-op suspicious prevertebral linvasion^a^		
No	55	83.3%
Yes	11	16.7%
Pre-op positive cervical nodes		
No	48	72.7%
Yes	18	27.3%

^a^Pre-operativesuspicious was judged by preoperative CT scan

### Post-operative pathologic findings

The entire cohort of cases had recurrent advanced local disease (rT4). Specifically, 21.2% of patients (14/66) had skin invasion, and 36.4% (26/66) and 16.7% (11/66) had microscopic invasion of the carotid artery and pre-vertebral fascia by frozen section and post-operative pathology.

### Complications

The overall complication rate was 39.4% (16/66), and no peri-operative deaths occurred. Flap loss occurred in 2 patients, and the incidence of fistulas and anastomosis strictures was 15.6% (10/66) and 13.6% (9/66), respectively. Other complications included a hematoma (*n* = 1) and wound infection (*n* = 2). The incidence of severe complications (flap loss, strictures, and fistulas) was 22.7% (15/66). Univariate analysis of the characteristics related to severe complications is presented in Table 2. The time interval between radiation and salvage surgery ≤6 months and previous concurrent chemotherapy or targeted therapy were risk factors of severe complications (Table 2).

**Table 2:** Binary logistic regression of factors for oral diet recovery

Characteristics	RR(95%Cl)	*P*-value
Age in years	0.486(0.108–20,178)	0.346
> 60		
≤ 60		
Previous treatment	1.043(0.330–3.295)	0.942
1 course without surgery		
1 course with surgery		
Multipl ecourses		
Dose of radiation	0.212(0.014–3.175)	0.261
> 60Gy		
≤ 60Gy		
Time interval between radiation and salvage surgery	10.224(1.455–71.839)	**0.019**
≤ 6		
> 6		
Previous chemo- or targeted therapy	0.129(0.026–0.646)	**0.013**
No		
Yes		
Skininvasion	0.100(0.010–1.022)	0.052
No		
Yes		
Intra-op carotid artery invasion^a^	0.274(0.030–2.491)	0.250
No		
Yes		
Pre-op prevertebral invasion	10.871(0.778–157.952)	0.076
No		
Yes		

^a^intra-op carotid artery invasion was judged by intraoperative detection

### Survival

The follow-up period was between 12 and 60 months, and the median follow-up period was 38 months for all living patients. Forty-one deaths occurred in the group.

The median survival time was 12 months, and the 1- and 2-year overall survival rates of the entire cohort were 50.4% and 37.7%, respectively.

**Fig. 1:**
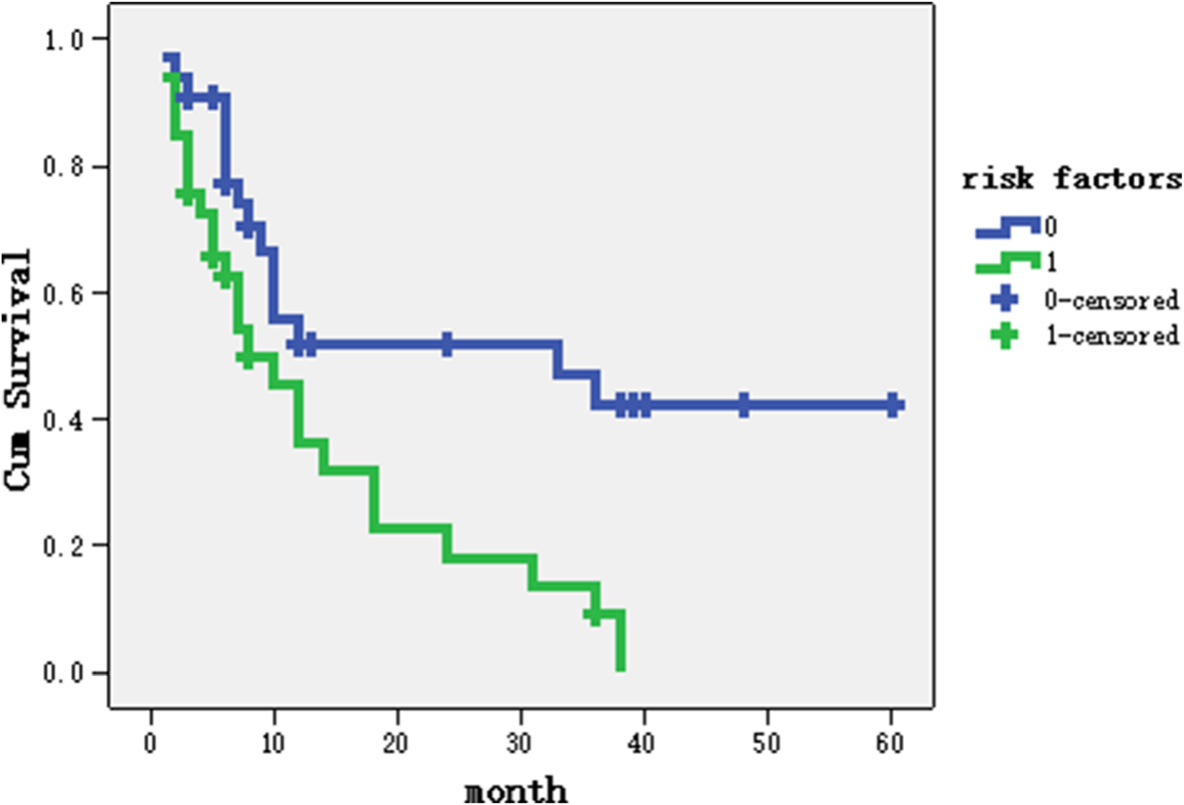
Survival rate of patients with and without two risk factors

Possible prognostic factors (age, primary site, previous treatment, time interval between radiation and salvage surgery, previous chemo- or targeted therapy, skin invasion pre- and intra- operative carotid artery or prevertebral invasion and positive lymph node) were including in univariate analysis (Table 3). The statistically significant factors (time interval between radiation and salvage surgery, microscopic carotid artery invasion, and pre-op pre-vertebral invasion) in univariate analysis were included in the multivariate analysis by Cox regression. The time interval between radiation and salvage surgery and microscopic carotid artery invasion were identified as independent prognostic factors (Table 4). The 3-year overall survival rates of patients with (*n* = 33) and without (*n* = 33) these two risk factors were 9.1% and 47.2%, respectively (*p* = 0.007), and the median survival rates of patients with and without two risk factors were 8 and 33 months, respectively (Fig. 1).

**Table 3:** Univariate analysis of prognostic factors

Characteristics	n	2-year overall survival rate	*P*-value
Age in years			0.285
> 60	30	42.2%	
≤ 60	36	33.9%	
Primary			0.218
Larynx	19	22.3%	
Hypo-pharynx	40	42.1%	
Cervicalesophagus	7	57.1%	
Previous treatment			0.692
1 course without surgery	30	30.9%	
1 course with surgery	26	43.0%	
Multiple courses	10	46.7%	
Time interval between radiation and salvage surgery			**0.065**
≤ 6	14	11.3%	
> 6	52	41.6%	
Previous chemo- or targeted therapy			0.579
No	46	40.3%	
Yes	20	31.4%	
Skin invasion			0.135
No	52	41.9%	
Yes	14	19.8%	
Pre-op suspicious carotid artery invasion^a^			**0.009**
No	53	42.8%	
Yes	13	8.5%	
Intra-op carotid artery invasion^b^			**0.000**
No	40	54.3%	
Yes	26	6.2%	
Pre-op suspicious prevertebral invasion^a^			**0.001**
No	55	46.0%	
Yes	11	0	
Intra-op prevertebral invasion^b^			0.327
No	39	44.1	
Yes	27	24.4	
Pre-op positive cervical nodes			0.834
No	48	38.4%	
Yes	18	34.4%	

^a^Pre-operative suspicious was judged by preoperative CT scan

^b^intra-op carotid artery invasion was judged by intraoperative detection

**Table 4:** Multivariate analysis for prognostic factors by Cox regression

Characteristics	2-year overall survival rate	HR(95%Cl)	*P*-value
Time interval between radiation and salvage surgery (months)		2.527 (1.219–5.242)	**0.013**
≤ 6	11.3%		
> 6	41.6%		
Intra-op carotid artery invasion		3.643 (1.593–8.329)	**0.002**
No	54.3%		
Yes	6.2%		
Pre-op pre vertebral invasion		0.643 (0.265–1.559)	0.328
No	46.0%		
Yes	0

## Discussion

Radiotherapy with chemotherapy is considered the mainstay treatment for squamous cell carcinoma of the head and neck; however, effective treatment is lacking for persistent or recurrent locally advanced lesions. The standard first-line palliative chemotherapy is used with a median survival of 6–9 months. Salvage surgery was associated with a 12-month survival for the entire group, especially the 33 patients without the 2 risk factors.

Traditionally, salvage surgery for advanced squamous cell carcinoma of the upper respiratory and digestive tracts also has a poor prognosis. Goodwin reported that the 2-year survival rate of salvage surgery for stage IV HNSCC was <25%, and only 30% of patients reported a satisfactory QOL [4]. TPLE overall prolonged the median survival time from 3.8–12 months compared with supportive care, which is meaningless considering the high risk of salvage surgery. As we found in the current study, select patients who did not have two risk factors could achieve a high 3-year survival rate of 47.2%, and the median survival of 33 months was similar to naïve-treatment patients with the same stage. Our results revealed that surgical salvage could still help such late-stage patients.

The time interval between radiation and salvage surgery was a significant factor related to survival and severe complications. The poor prognostic patients developed rT4 tumors <6 months after radiation. The possible reasons were as follows: (1) the cancer was resistant to radiation; or (2) the tumor progressed quickly after recurrence. Each condition indicates that these tumors exhibit aggressive biological behavior, and salvage surgery is not a promising strategy to treat these tumors. This finding is similar to that reported in other studies [2–4]. Some authors [5–7] have considered that a short disease interval (DFI) is a significant negative tumor factor, as well as a faster growth rate or greater resistance to index treatment, making salvage therapy more problematic [8–11].

Microscopic carotid artery invasion was a prognostic factor in the current study. Although clinical rT4b cases were excluded according to pre-operative evaluation, microscopic invasion of the carotid artery was still detected intra-operatively by frozen section; however, this risk factor seemed difficult to be distinguished before surgery, which was still a problem we faced. Tumor and scar tissue were difficult to distinguish by imaging examination and more accurate preoperative evaluation is still needed for recurrent disease.

In the current study, the negative factors related to oncologic and functional improvement included a shorter interval between radiation and salvage surgery, microscopic carotid artery invasion, and previous concurrent chemotherapy or targeted therapy. These factors were similar to those reported in previous studies on salvage surgery for recurrent HNSCC regardless of previous treatment or recurrence status [1, 12–15]. This result demonstrated that selected cases, even very locally advanced recurrent head and neck cases, could also be salvaged by surgery. Patients are likely to benefit from salvage TPLE.

Considering these results, we recommend individualized assessment and multi-disciplinary decision of each patient. For patient who do not have any adverse factors of prognosis and post-operative QOL, salvage surgery is a good choice.

## Conclusions

Salvage TPLE is likely to have significant survival and functional benefit to select patients with previously irradiated, recurrent T4 cancer of the larynx, hypopharynx, and cervical esophagus. The adverse factors include a short interval between irradiation and salvage surgery, microscopic carotid artery invasion, and previous concurrent chemotherapy or targeted therapy.

## References

[1] Kowalski LP, Carvalho AL (2000). Natural history of untreated head and neck cancer. Eur J Cancer.

[2] Putten L, Bree R, Doornaert PA, Buter J, Eerenstein SE, Rietveld DH, Kuik DJ, Leemnas CR (2015). Salvage surgery in post-chemoradiation laryngeal and hypopharyngeal carcinoma: outcome and review. Acta Otorhinolaryngol Ital.

[3] Tan HK, Giger R, Auperin A, Bourhis J, Janot F, Temam S (2010). Salvage surgery after concomitant chemoradiation in head and neck squamous cell carcinomas - stratification for post salvage survival. Head Neck..

[4] Goodwin WJ (2000). Salvage surgery for patients with recurrent squamous cell carcinoma of the upper aerodigestive tract: when do the ends justify the means?. Laryngoscope.

[5] Zafereo ME, Hanasono MM, Rosenthal DI, Sturgis EM, Lewin JS, Roberts DB, Weber RS (2009). The role of salvage surgery in patients with recurrent squamous cell carcinoma of the oropharynx. Cancer.

[6] Stell PM (1991). Time to recurrence of squamous cell carcinoma of the head and neck. Head Neck..

[7] Liao CT, Chang JT, Wang HM, Ng SH, Hsueh C, Lee LY, Lin CH, Huang SF, Cheng AJ, Yen TC (2008). Salvage therapy in relapsed squamous cell carcinoma of the oral cavity: how and when?. Cancer.

[8] Ho AS, Kraus DH, Ganly I, Lee NY, Shah JP, Morris LG (2014). Decision making in the management of recurrent head and neck cancer. Head Neck..

[9] Argiris A, Li Y, Forastiere A (2004). Prognostic factors and long-term survivorship in patients with recurrent or metastatic carcinoma of the head and neck. Cancer.

[10] Colevas AD (2006). Chemotherapy options for patients with metastatic or recurrent squamous cell carcinoma of the head and neck. J Clin Oncol.

[11] Fury MG, Pfister DG (2011). Current recommendations for systemic therapy of recurrent and/or metastatic head and neck squamous cell cancer. J Natl Compr Cancer Netw.

[12] Ganly I, Patel SG, Matsuo J, Singh B, Kraus DH, Boyle JO, Wong RJ, Shaha AR, Lee N, Shah JP (2006). Results of surgical salvage after failure of definitive radiation therapy for early-stage squamous cell carcinoma of the glottic larynx. Arch Otolaryngol Head Neck Surg.

[13] Holsinger FC, Funk E, Roberts DB, Diaz EM (2006). Conservation laryngeal surgery versus total laryngectomy for radiation failure in laryngeal cancer. Head Neck.

[14] Pivot X, Niyikiza C, Poissonnet G, Dassonville O, Bensadoun RJ, Guardiola E, Foa C, Benezery K, Demard F, Thyss A, Schneider M (2001). Clinical prognostic factors for patients with recurrent head and neck cancer: implications for randomized trials. Oncology.

[15] Li M, Lorenz RR, Khan MJ, Burkey BB, Adelstein DJ, Greskovich JF, Koyfman SA, Scharpf J (2013). Salvage laryngectomy in patients with recurrent laryngeal cancer in the setting of nonoperative treatment failure. Otolaryngol Head Neck Surg.

